# Sesamin Metabolites Suppress the Induction of Cellular Senescence

**DOI:** 10.3390/nu15071627

**Published:** 2023-03-27

**Authors:** Chie Araki, Daisuke Takemoto, Yoshinori Kitagawa, Norifumi Tateishi, Tomohiro Rogi, Takayuki Izumo, Shimpei Kawamoto, Hiroshi Shibata, Eiji Hara, Masaaki Nakai

**Affiliations:** 1 Institute for Health Care Science, Suntory Wellness Limited, Kyoto 619-0284, Japan; 2 Departments of Molecular Microbiology, Research Institute for Microbial Diseases, Osaka University, Osaka 565-0871, Japan

**Keywords:** sesamin, episesamin, SC1, EC1-2, cellular senescence, mitochondria

## Abstract

Cellular senescence induces inflammation and is now considered one of the causes of organismal aging. Accumulating evidence indicates that age-related deterioration of mitochondrial function leads to an increase in reactive oxygen species (ROS) and DNA damage, which in turn causes cellular senescence. Thus, it is important to maintain mitochondrial function and suppress oxidative stress in order to inhibit the accumulation of senescent cells. Sesamin and its isomer episesamin are types of lignans found in sesame oil, and after being metabolized in the liver, their metabolites have been reported to exhibit antioxidant properties. However, their effects on cellular senescence remain unknown. In this study, the effects of sesamin, episesamin, and their metabolites SC1 and EC1-2 on replicative senescence were evaluated using human diploid lung fibroblasts, and TIG-3 cells. The results showed that sesamin and episesamin treatment had no effect on proliferative capacity compared to the untreated late passage group, whereas SC1 and EC1-2 treatment improved proliferative capacity and mitigated DNA damage of TIG-3 cells. Furthermore, other cellular senescence markers, such as senescence-associated secretory phenotype (SASP), mitochondria-derived ROS, and mitochondrial function (ROS/ATP ratio) were also reduced by SC1 and EC1-2 treatment. These results suggest that SC1 and EC1-2 can maintain proper mitochondrial function and suppress the induction of cellular senescence.

## 1. Introduction

When normal cells are stimulated by DNA damage, cellular senescence is induced and irreversible growth arrest occurs, making cell division impossible [1,2]. Thus, cellular senescence contributes to living organisms as a mechanism to suppress the carcinogenesis that could be induced by DNA damage [1,2,3]. Characteristic features accompanying cellular senescence are stable cell cycle arrest, flat and enlarged cell shapes [4,5] induction of p16^INK4a^ and p21^Waf1/Cip1^ [6,7], and secretion of various pro-inflammatory factors, called senescence-associated secretory phenotype (SASP) [8,9].

The increase in oxidative stress with age [10,11] makes cellular senescence more likely, and senescent cells accumulate with age [12]. Increased SASP factors secreted by senescent cells may promote chronic inflammation, accelerating age-related functional declines and diseases [1]. In fact, the removal of p16^Ink4a^-positive senescent cells from aged mice reportedly delays age-related dysfunction and extends a healthy life span [13,14,15]. These pieces of evidence suggest that the accumulation of senescent cells in vivo is involved in the onset of aging and age-related diseases.

It is reported that cells treated with antimycin inhibit mitochondrial function, increase reactive oxygen species (ROS) and lead to cellular senescence [16]. Therefore, suppression of ROS production by maintaining mitochondrial function may contribute to the prevention of cellular senescence induction. In senescent cells, metabolic function is also in an active state [17] and mitochondrial mass is known to be increased with the induction of cellular senescence [17,18], which increases ATP production [17]. Since senescent cells have a high energy demand due to increased cell size and SASP production, large amounts of ATP are required [17]. In senescent cells, increased mitochondria-derived ROS [19,20,21], decreased mitophagy [22,23,24], and mitochondrial morphological abnormalities [22] have also been observed. Thus, even after cellular senescence is induced, functionally compromised mitochondria further promote the induction and maintenance of cellular senescence, suggesting that reducing mitochondria-derived ROS is important to inhibit senescent cell accumulation.

Several cellular senescence induction models have been reported to evaluate the anti-senescence effects of compounds, including oxidative DNA damage, oncogene activation, and replicative senescence [25]. The main cause of replicative senescence, aside from telomere shortening, is thought to be intracellular oxidative stress [26]. Though stress-induced senescence is caused by exogenous stimulation such as activation of oncogenes, replicative senescence is caused by endogenous stimulation, which is mainly related to mitochondrial function [26]. In replicative senescence, the cell cycle regulators p16^INK4a^ and p21^Waf1/Cip1^ are activated and stop cell division [27]. In addition, mitochondrial mass [18] and mitochondrial oxygen consumption [17] increase in replicative senescence.

Sesamin, a lignan compound found in sesame seeds and oil, is partly epimerized and converted to episesamin during the refining of non-roasted sesame seed oil. Sesamin and episesamin are metabolized in the liver and their metabolites exhibit antioxidative properties [28,29,30]. It has also been reported that sesamin shows anti-aging effects, such as reducing mitochondrial oxidative stress [31], improvement of endurance capacity in mice through slowing the decline of mitochondrial function by inhibiting NADPH oxidase (NOX) [32], and improving life spans in Drosophila [33]. However, the effects of sesamin, episesamin, and their metabolites SC1 and EC1-2 on cellular senescence remain unclear. In this study, we aimed to clarify the effects of sesamin, episesamin, and their metabolites SC1 and EC1-2 on replicative senescence.

## 2. Materials and Methods

### 2.1. Cell Culture

A TIG-3 cell line (ATCC) isolated from human fetal lung fibroblasts was used in the present study. TIG-3 cells are thought to show replicative senescence at a population doubling level (PDL) of 70 or higher [34]. The cells were cultured in an incubator at 37 °C and 5% CO_2_ in Dulbecco’s Modification of Eagle’s Medium (DMEM) supplemented with 10% FBS and 1% Antibiotic Antimycotic Mixed Stock Solution (Nacalai Tesque, Kyoto, Japan). Cells were seeded at 2 × 10^5^ cells/2 mL medium/well when the passage was conducted after 3 days, and 1 × 10^5^ cells/2 mL medium/well when the passage was conducted after 4 days. At PDL70 and higher, the passage was conducted once a week, and the seeding density was 1 × 10^5^ cells/2 mL medium/well. Cells whose PDLs are around 40 were used as “early passage group” cells. Cells were treated with the test substance from the start of the culture (cells around PDL40), and the test substance was added regularly until proliferation ceased (“late passage group” cells).

### 2.2. Test Compounds

For sesamin and episesamin, raw materials isolated from sesame were refined and separated with HPLC. The sesamin metabolite SC1 [(7α,7′α,8α,8′α)-3′,4′-methylenedioxy-7,9′:7′,9-diepoxylignane-3,4-diol] and episesamin metabolite EC1-2 [(7α,7′β,8α,8′α)-3,4-methylenedioxy-7,9′:7′,9-diepoxylignane-3′,4′-diol] were prepared by chemical synthesis. The metabolites were isolated and refined as single compounds, and their chemical structures were confirmed using nuclear magnetic resonance [35,36]. In this study, EC1-1 [(7α,7′β,8α,8′α)-3′,4′-methylenedioxy-7,9′:7′,9-diepoxylignane-3,4-diol], another metabolite of episesamin, was not used for the experiment because of less amount. DMSO was used for the solvent, with 0.1% contained in the culture medium.

### 2.3. γH2AX Immunostaining

γH2AX immunostaining in TIG-3 cells was conducted using a DNA Damage Detection Kit–γH2AX–Green (Dojindo Laboratories, Kumamoto, Japan), with assessments completed in accordance with the manufacturer’s instructions. Cells were seeded at 1 × 10^4^ cells for the early passage group (PDL 43) and 2 × 10^4^ cells for the late passage group (PDL 69–70). After culturing for 2 days, the cells were fixed with 4% PFA. After adding 0.5% Triton-X, 3% BSA was added. Next, 250 μL of primary antibodies were added, and the cells were left to sit overnight at 4 °C. They were then treated with a secondary antibody, after which 500 ng/mL DAPI was added, and images were taken and analyzed with a BZ-X Analyzer (KEYENCE, Osaka, Japan).

### 2.4. Western Blotting

Early passage group (PDL 44) and late passage group (PDL 73–74) were collected in solution in which Halt™ Protease & Phosphatase Inhibitor Cocktail (Thermo Fisher Scientific, Waltham, MA, USA) was added to RIPA buffer (FUJIFILM Wako Pure Chemical, Osaka, Japan) and incubated for 30 min on ice. Next, cells were lysed in a sonication system and centrifuged at 10,000× *g* and 4 °C for 20 min, and the supernatant was collected.

The protein concentration was measured and adjusted, 5 μg were separated in SuperSep™ Ace, 17-well, 10–20% gel (FUJIFILM Wako Pure Chemical, Osaka, Japan), and blotted on a PVDF membrane (Bio-Rad, Hercules, CA, USA). In accordance with the manufacturer’s instructions, the membrane was incubated together with primary antibody ARPC5/p16 ARC (ab51243, Abcam, Cambridge, UK) overnight at 4 °C. After washing several times in TBS containing 0.05% Tween-20, incubation was continued for at least one hour at room temperature together with HRP-labeled secondary antibody which corresponds to the primary antibody. After detection of the bands, antibodies were stripped using a stripping solution (193-16375, FUJIFILM Wako Pure Chemical, Osaka, Japan) for 10 min at room temperature. After washing, then the membrane was incubated with HRP-labeled antibody against beta-Actin (ab20272, Abcam, Cambridge, UK) for one hour at room temperature. Specific bands were detected using enhanced chemiluminescence. Chemiluminescence was imaged using a FusionFx (Vilver, Collegien, France), and the relative intensity of the bands was measured.

### 2.5. qRT-PCR

To determine the mRNA expression level of the early passage group (PDL 42–46) and late passage group (PDL 69–72), all RNA was isolated using QIAzol (Qiagen, Venlo, The Netherlands) according to the manufacturer’s instructions. RNA concentration was measured with a NanoDrop™ UV spectrophotometer (Thermo Fisher Scientific, Waltham, MA, USA). cDNA was synthesized using High-Capacity cDNA Reverse Transcription Kit with RNase Inhibitor (Thermo Fisher Scientific, Waltham, MA, USA). TB Green^®^ Premix Ex Taq™ II (Takara, Siga, Japan) was used with the following probes and primers: IL-1β (forward, 5′-CTGTCCTGCGTGTTGAAAGA-3′;reverse, 5′-TTGGGTAATTTTTGGGATCTACA-3′;probe); IL-8 (forward, 5′-AAGGAAAACTGGGTGCAGAG-3′;reverse, 5′-ATTGCATCTGGCAACCCTAC-3′;probe); β-actin (forward, 5′-TGGATCAGCAAGCAGGAGTATG-3′;reverse, 5′-GCATTTGCGGTGGACGAT-3′;probe). The level of each SASP mRNA was normalized to the level of β-actin.

### 2.6. Mitochondrial Superoxide Production

To evaluate the mitochondrial function, the early passage group (PDL 31) and late passage group (PDL 73–74) were used. Mitochondrial superoxide production was evaluated by Mito SOX Red (Thermo Fisher Scientific, Waltham, MA, USA) in accordance with the manufacturer’s instructions. Cells were seeded at 3 × 10^4^ cells on 96-well plates, and after culturing for one day, they were stained with Mito SOX Red. Mito SOX Red was evaluated with a fluorescence microscope (BZ-X Analyzer).

### 2.7. Mitochondrial DNA Contents

Mitochondrial DNA was extracted using a kit (Takara, Siga, Japan) in accordance with the manufacturer’s instructions. DNA samples were analyzed with a real-time PCR system. The primers used were the Human Mitochondrial DNA (mtDNA) Monitoring Primer Set (Takara, Siga, Japan).

### 2.8. Oxygen Consumption Rate (OCR) Measurements

Cells were seeded at 1.5 × 10^4^ cells on a dedicated plate, and after culturing for one day, they were switched to an analysis medium. OCR was measured with an XF analyzer (Agilent Technologies, Santa Clara, CA, USA). Using a Seahorse XF Cell Mito Stress Test kit (Agilent Technologies, Santa Clara, CA, USA), OCR was examined according to the manufacturer’s instructions. Cellular ATP production was estimated with a sequential blockade of the mitochondrial respiratory chain by treating cells with oligomycin, FCCP (carbonylcyanide 4-[trifluoromethoxy] phenylhydrazone), rotenone, and antimycin A. After the analysis was completed, nuclei were stained with Hoechst (Nacalai Tesque, Kyoto, Japan), and the cells were photographed with a fluorescence microscope BZ-X Analyzer. The cells were counted, and then the OCR measurement values were normalized to the number of cells or the number of mitochondrial DNA copies. When measuring OCR, inhibitor treatment was conducted with oligomycin (1.5 μM), FCCP, antimycin, and rotenone (500 nM each).

### 2.9. Statistical Analysis

Statistical analyses were conducted using Dunnett’s test (*; *p* < 0.05, **; *p* < 0.01).

## 3. Results

### 3.1. Maintenance of Proliferative Capacity of TIG-3 Cell with SC1 and EC1-2

TIG-3 cells were cultured for approximately 70 days, and the PDL was measured at the time of growth arrest in an untreated late-passage group (Table 1). When treated with 1 μM SC1 and EC1-2, the results showed a significant increase in PDL compared with the late passage group. There was no significant change compared with the late passage group when treated with 1 μM sesamin, 1 μM episesamin, 100 nM SC1, or 100 nM EC1-2.

**Figure 1:**
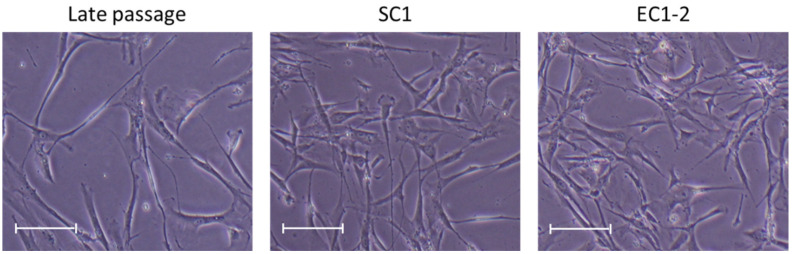
Effects of SC1 and EC1-2 on size of TIG-3 replicative senescence cells. Representative images show the size of each TIG-3 cell at PDL 73–74. Scale bar = 200 µm.

Regarding cell morphology, the cells in the late passage group had become flatter and larger, but these changes were suppressed by 1 μM SC1 and EC1-2 treatment (Figure 1).

### 3.2. Inhibition of DNA Damage with SC1 and EC1-2

**Figure 2:**
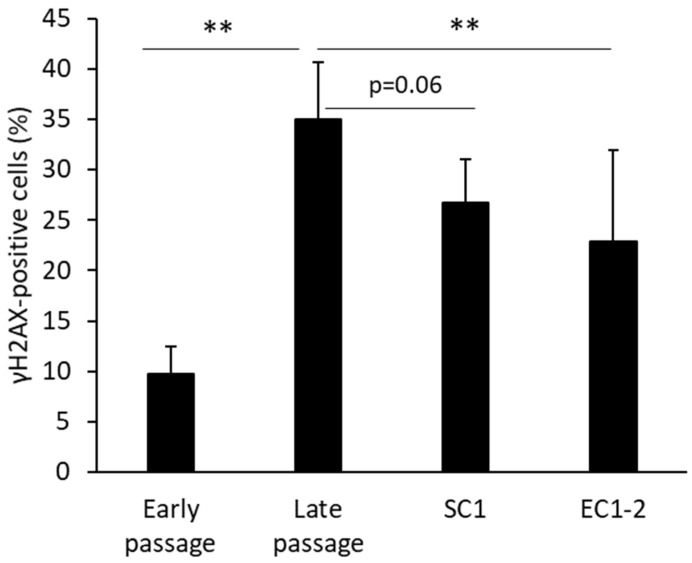
Effects of SC1 and EC1-2 on the ratio of γH2AX positive cells. γH2AX-positive cells and γH2AX-negative cells are distinguished by measuring the fluorescence intensity of γH2AX immunostaining. Data are reported as means ± SD of three independent experiments. **; *p* < 0.01 vs. Late passage group by Dunnett’s test.

When the extent of cellular DNA damage was measured (Figure 2), the ratio of γH2AX-positive cells was increased significantly in the late passage group compared with the early passage group. Treatment with 1 μM EC1-2 showed significant suppression of γH2AX-positive cells, and treatment with 1 μM SC1 showed a trend of suppression (*p* = 0.06) compared to the late passage group. 

### 3.3. Inhibition of a Cellular Senescence Marker with SC1 and EC1-2

**Figure 3:**
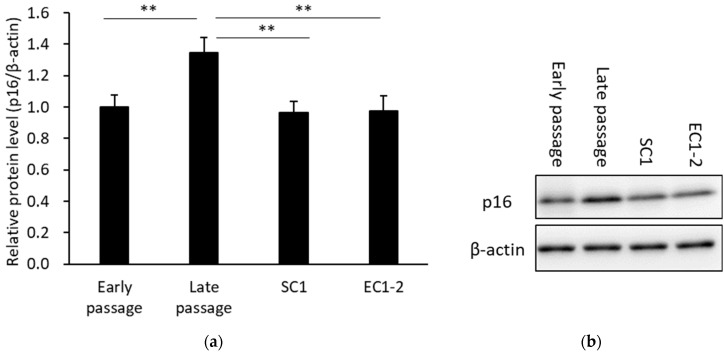
Effects of SC1 and EC1-2 on the cellular senescence marker p16 (**a**). The relative protein level of p16 normalized with β-actin is evaluated by western blotting. Representative western blot images of p16 and β-actin are shown (**b**). Data were reported as means ± SD of three independent experiments. **; *p* < 0.01 vs. Late passage group by Dunnett’s test.

The results of western blotting for the p16, a cellular senescence marker, are shown in Figure 3. When compared with the early passage group, p16 protein mass was significantly increased in the late passage group. The increase in p16 protein was suppressed with 1 μM SC1 or EC1-2 treatment. These results confirmed that the induction of replicative cellular senescence is suppressed by SC1 and EC1-2. On the other hand, the treatment of SC1 and EC1-2 did not affect the p16 expression levels in the cellular senescence induced by the genotoxic doxorubicin (see Figure S1). Original gel images of the western blot in Figure 3b are shown in Figure S2.

### 3.4. Inhibition of SASP with SC1 and EC1-2

**Figure 4:**
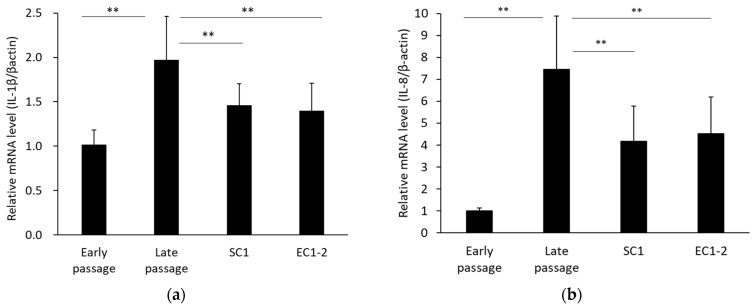
Effects of SC1 and EC1-2 on SASP. The relative mRNA levels of IL-1β (**a**) and IL-8 (**b**) normalized with β-actin were evaluated with qRT-PCR. Data were reported as means ± SD of three independent experiments. ** *p* < 0.01 vs. Late passage group by Dunnett’s test.

Senescence-associated secretary phenotypes (SASP) were measured (Figure 4). Compared with the early passage group, mRNA expression of IL-1β (a) and IL-8 (b) was significantly increased in the late passage group. In contrast, with 1 μM SC1 and EC1-2 treatment, IL-1β and IL-8 were significantly suppressed compared with the late passage group.

### 3.5. Inhibition of Superoxides in Mitochondria with SC1 and EC1-2

**Figure 5:**
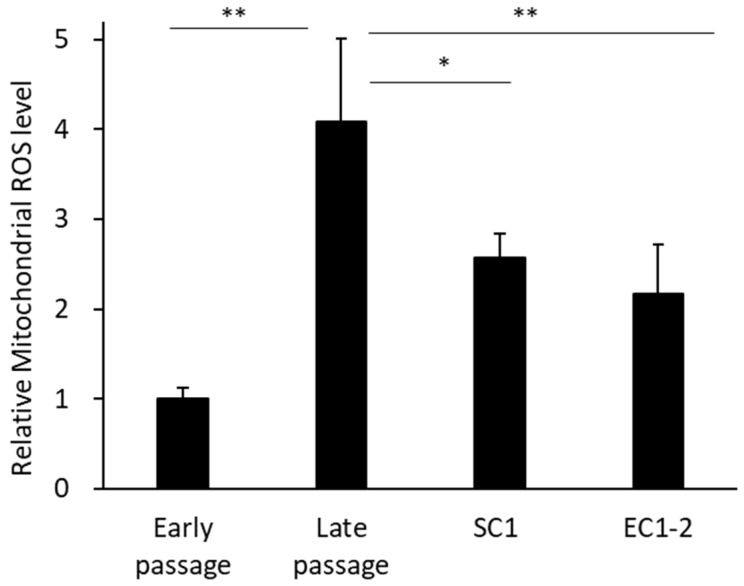
Effects of SC1 and EC1-2 on superoxides in mitochondria. Superoxide production in mitochondria is evaluated with Mito SOX Red staining. Data are reported as means ± SD of three independent experiments. *; *p* < 0.05, **; *p* < 0.01 vs. Late passage group by Dunnett’s test.

Superoxides in mitochondria were measured (Figure 5). Compared with the early passage group, the amount of ROS in mitochondria was significantly increased in the late passage group. In contrast, the amount of ROS in mitochondria was significantly suppressed by 1 μM SC1 and EC1-2 treatment compared with the late passage group.

### 3.6. Improvement of Mitochondrial Function with SC1 and EC1-2

**Figure 6:**
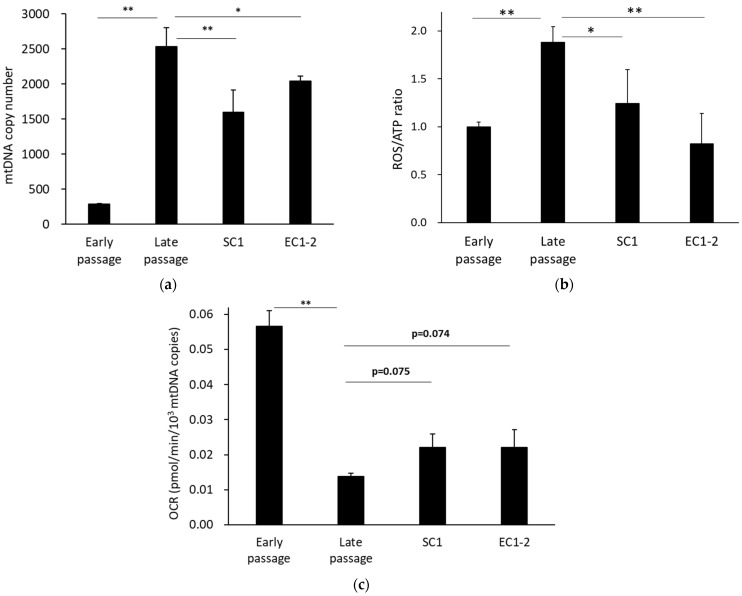
Effects of SC1 and EC1-2 on the number of mitochondrial DNA copies. The number of mtDNA copies in TIG-3 cells analyzed by qPCR (**a**). Effects of SC1 and EC1-2 on the ROS/ATP ratio. The ROS/ATP ratio was calculated with the superoxide production levels in mitochondria and the ATP production rate was calculated from the oxygen consumption rate (OCR) per cell (**b**). Effects of SC1 and EC1-2 on ATP production. ATP production rate calculated from OCR was corrected for the number of mitochondrial DNA copies (**c**). Data are reported as means ± SD of three independent experiments. *; *p* < 0.05, **; *p* < 0.01 vs. Late passage group by Dunnett’s test.

The number of mitochondrial DNA copies in cells was measured (Figure 6a), and there were significantly more mitochondrial DNA copies in the late passage group than in the early passage group. The results also showed that the number of mitochondrial DNA copies was significantly suppressed with 1 μM SC1 and EC1-2 treatment. For mitochondrial function, the ROS/ATP index (Figure 6b), which is the ratio of the amount of ROS in the mitochondria and the ATP production per cell, was calculated. This can be interpreted as the amount of ROS produced when one ATP is synthesized. The ROS/ATP ratio was significantly increased in the late passage group compared with the early passage group. In contrast, the ROS/ATP ratio was significantly suppressed with 1 μM SC1 and EC1-2 treatment. In addition, when the ATP production rate corrected for the number of mitochondrial DNA copies was calculated as ATP production capacity per mitochondria (Figure 6c), it was significantly lower in the late passage group than in the early passage group, and a trend for suppression of the decrease was shown with 1 μM SC1 and EC1-2 treatment. These results suggest that mitochondrial function is improved with SC1 and EC1-2 treatment.

## 4. Discussion

It has been reported that sesamin reduces mitochondrial oxidative stress [31,32], slowing the decrease in function associated with aging in Drosophila and increasing their life span [33]. Although there are few reports of the efficacy of episesamin, it has been reported that, after sesamin and its isomer episesamin are metabolized in the liver, their metabolites SC1 and EC1-2 show an antioxidative effect [28,29,30]. We hypothesized that SC1 and EC1-2, which have an anti-oxidative function, may inhibit DNA damage, thought to be the fundamental mechanism of senescence, and the induction of cellular senescence by maintaining the mitochondrial functions that decline with age. In this study, sesamin, episesamin, and their metabolites SC1 and EC1-2 were evaluated in a replicative senescence model.

In this replicative senescence experiment, human fetal lung fibroblast TIG-3 cells were used. TIG-3 cells are reported to show the phenotypes of replicative senescence at PDL of 70 or higher [34]. Similar to a previous study, the growth of TIG-3 cells was arrested at PDL73.2. It has also been reported that senescent cells are in a metabolically activated state and show increases in the mitochondrial mass and ATP production rate [17,18]. A trend towards increased ATP production rate was also observed in TIG-3 late passage group in the present study. Replicative senescence is thought to be caused by endogenous stimulation, particularly increased ROS from mitochondria [26]. In fact, it was observed in the present study that, in TIG-3 cells, mitochondrial ROS increased and the ROS/ATP ratio increased just around the timing of growth arrest (around PDL of 73). However, DNA damage indexed by γH2AX, which is thought to be the cause of cellular senescence, is observed around PDL of 69 before growth arrest. Thus, mitochondrial function decreases gradually during the replicative senescence process prior to growth arrest, suggesting that cellular senescence has been induced by increasing the amount of ROS in mitochondria and subsequent DNA damages, although there is no direct evidence that ROS production was also increased during the transition phase of cellular senescence.

In cellular senescence, DNA damage results in an increase in the expression level of the p16 protein, which is a cell cycle inhibitor [1,2,6,7] and is thought to be a typical marker of senescent cells [37]. It has been reported that the selective elimination of p16^Ink4a^-positive senescent cells could extend a healthy lifespan and improve the various defects associated with aging [13,14,15]. In the present study, the amount of p16 protein increased with DNA damage in replicative senescence and was suppressed by SC1 and EC1-2 treatment. The above results suggest that the induction of cellular senescence is possibly inhibited by SC1 and EC1-2 as a result of the suppression of DNA damage due to ROS derived from impaired mitochondria. In the Drosophila senescence-accelerated model, Sod1n1 mutant adults fed with sesamin showed a decreased frequency of nuclei exhibiting DNA damage foci detected by anti-γH2AvD immunostaining and increased mRNA levels of two DNA repair factors (Gadd45 and CG9272) [33]. Sesamin was found to be absorbed and metabolized to SC1 in Drosophila adults. Therefore, in replicative senescent TIG-3 cells, SC1 and EC1-2 could also induce these DNA repair genes which contribute to reducing the induction of the p16 protein.

It has also been demonstrated that, in senescent cells, chromatin fragments derived from genomic DNA accumulate in the cytoplasm as a result of the continuous DNA damage response (DDR) caused by ROS, and activation of the cGAS–STING pathway, which is a nucleic acid sensor, plays an important role in SASP gene expression [38]. IL-1β, a kind of the SASP, is reported to be a powerful inducer of IL-8 expression [39], and the analysis of SASP gene expression demonstrated that increased IL-8 and IL-1β expressions are suppressed with SC1 and EC1-2. It is thought that the suppression of DNA damage by SC1 and EC1-2 inhibited the accumulation of DNA fragments and the expression of SASP factors. Higher levels of SASP factors secreted by senescent cells promote chronic inflammation, and this may accelerate functional declines associated with aging and age-related diseases [1]. SC1 and EC1-2 might have the potential to delay the functional declines which depend on the SASP expression with aging.

In the present study, the ROS/ATP ratio [40,41] was used as an indicator of mitochondrial function. ROS/ATP ratio was increased in senescent TIG-3 cells, which suggests that mitochondrial function declined in cellular senescence. Moreover, SC1 and EC1-2 improved the ROS/ATP ratio significantly. These results suggest that maintaining mitochondrial function with SC1 and EC1-2 during the replicative senescence suppresses the promotion of cellular senescence.

Increased ROS due to the decline of mitochondrial function is considered to be a cause of the induction of cellular senescence [16], and the effects of SC1 and EC1-2 on mitochondrial function were therefore evaluated. The increased mitochondrial mass and ATP production rate in senescent cells likely occur to compensate for the higher energy demand from increased cell size and SASP production [17,18]. Increases in the number of mitochondrial DNA copies in the late passage group were suppressed by SC1 and EC1-2, but the ATP production rate was maintained. Since a correlation between mitochondrial DNA copy number and mitochondrial mass has been reported [42], the ATP production rate per mitochondrion was calculated by correcting the data on the ATP production rate per cell for the number of mitochondrial DNA copies. As a result, a significant decrease in ATP production rate per mitochondrion was observed in the late passage group compared with the early passage group. Contrarily, the ATP production rate per mitochondrion showed a tendency to improve with SC1 and EC1-2. Thus, the ATP production rate was considered to be maintained as a result of the improved ATP production efficiency per mitochondrion with SC1 and EC1-2.

A similar investigation was conducted using TIG-1 cells, which are also human fetal lung fibroblasts [43]. In replicative senescent TIG-1 cells, an increase in the amount of mitochondrial ROS per cell has been confirmed, as with TIG-3 cells [43]. It was also reported, however, that, in TIG-1 cells, a decline in mitochondrial function and an increase in mitochondrial ROS production occur after the completion of cellular senescence, where there is growth arrest [43]. Further studies are needed to determine whether ROS production associated with mitochondrial dysfunction is a prerequisite for the process of cellular senescence in a replicative senescence model.

In senescent cells, dysfunctional mitochondria cannot be eliminated from the mitochondrial network due to the impairment of mitochondrial fission, and mitophagy is impaired [44]. Decreased mitophagy could lead to an increase in mitochondria with declined function. According to the previous study, sesamin could improve autophagy by inhibiting mTOR [45]. Further investigation is needed to determine whether sesamin metabolites could induce autophagy to prevent cellular senescence.

In the present study, the sesamin and episesamin metabolites SC1 and EC1-2 showed inhibiting effects of cellular senescence. There are very few previous studies on the efficacy of EC1-2, but similar to SC1, a direct radical scavenging capacity has been reported [29]. It is possible that decreases in mitochondrial function are suppressed by the direct radical scavenging capacity of SC1 and EC1-2. The actions of SC1 and EC1-2 were similar in this study, and further investigation is required to determine which one shows a stronger effect against cellular senescence through radical scavenging capacity. Since upregulation of the antioxidant enzymes such as superoxide dismutase, glutathione peroxidase, and glutathione S-transferase by SC1 are reported [46,47], there is also a possibility that the increase of intracellular antioxidative activities is related to the suppression of cellular senescence. It was demonstrated that sesamin protected Drosophila adults against oxidative damage via stimulation of the Nrf2/Cnc-dependent transcription in the adult gut and brain [48]. Elucidation of the mechanisms of action with SC1 and EC1-2 is the next issue for future study.

## 5. Conclusions

The present study suggested, in a replicative senescence model using human cells, that SC1 and EC1-2, metabolites of sesamin and episesamin, may serve to maintain mitochondrial function appropriately, prevent DNA damage from mitochondria-derived ROS, and suppress the induction of cellular senescence in the aging process.

## Figures and Tables

**Table 1:** Effects of sesamin, episesamin, and their metabolites on proliferative capacity.

Treatment Group		PDL
Late passage	-	73.2 ± 0.44
Sesamin	1 μM	73.6 ± 0.47
Epiesamin	1 μM	73.6 ± 0.45
SC1	100 nM	73.4 ± 0.37
	1 μM	74.5 ± 0.57 *
EC1-2	100 nM	72.3 ± 0.76
	1 μM	74.5 ± 0.32 *

PDL40 human lung fibroblast TIG-3 cells were cultured in DMEM to which the above compounds were added. Cells were treated with the test substance from the start of culture (PDL40), and the test substance was added regularly until proliferation in the late passage group ceased PDL was calculated as log2 (D/D0), with D and D0 defined as the cell number at the time of harvesting and the time of seeding, respectively. The data are reported as means ± SD of three independent experiments. *; *p* < 0.05 vs. Late passage group by Dunnett’s test.

## Data Availability

Not applicable.

## Supplementary Materials

The following supporting information can be downloaded at: https://www.mdpi.com/article/10.3390/nu15071627/s1, Figure S1: Changes in the cellular senescence marker p16 with the treatment of NAC, SC1, and EC1-2 before DXR treatment. Figure S2: Original gel images of the western blot in Figure 3b.
