# Supplementary material for: Sesamin Metabolites Suppress the Induction of Cellular Senescence
Source: Nutrients. 2023 Mar 27;15(7):1627. doi: 10.3390/nu15071627 (PMC10096530; doi:10.3390/nu15071627)
Supplement: Supplementary file 1 [file nutrients-15-01627-s001.zip › nutrients-2263796-supplementary.pdf]

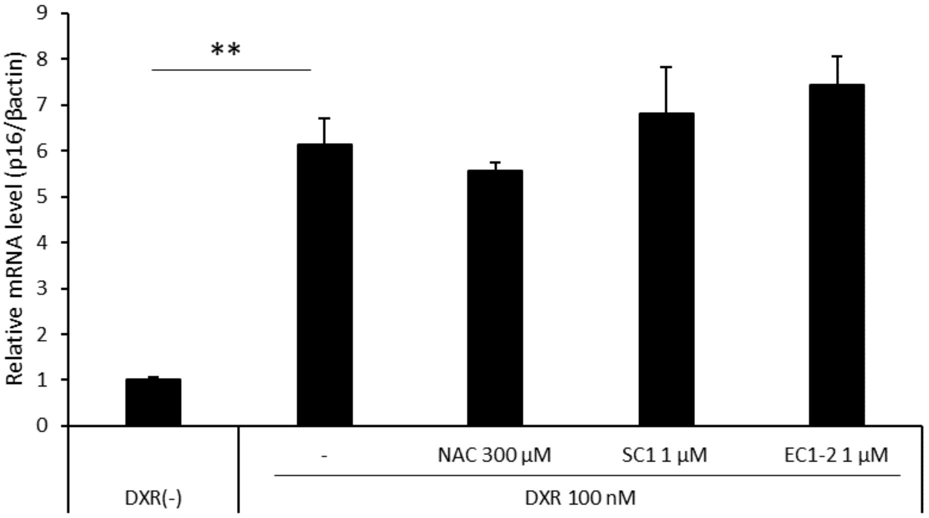

Figure S1. Changes in the cellular senescence marker p16 with treatment of NAC, SC1 and EC1-2 before DXR treatment. The level of mRNA expression was normalized to the level of β-actin. Data are reported as means ± SD of three independent experiments. **; $p < 0.01$ vs. DXR-induced senescence group by Dunnett's test.

Materials and Methods: TIG-3 cells were seeded at 4 × 104 cells/well in 6 wells, and N-acetylcysteine (NAC), SC1 and EC1-2 were added. After 4 hours, 100 nM doxorubicin was added, and the cells were cultured for 7 days. They were then switched to DMEM supplemented with 10% FBS and 1% Antibiotic Antimycotic Mixed Stock Solution and cultured for 3 days, after which the cells were collected. mRNA expression was measured by qRT-PCR used the following primers; p16 (forward, 5′- CCAACGCACCGAATAGTTACG -3′; reverse, 5′- GCGCTGCCCATCATCATG -3′; probe).

Results and Discussion: Doxorubicin (DXR), which breaks the DNA double strand, was added, and the actions of the antioxidant substances N-acetylcysteine (NAC), SC1, and EC1-2 on cellular senescence were investigated. Compared with no DXR treatment, p16 expression increased significantly by six-fold with DXR treatment. In a group treated with NAC, SC1 and EC1-2 before DXR treatment, there is no significant change compared with the DXR group. These results suggest that SC1 and EC1-2, similar to NAC, do not inhibit the induction of cellular senescence when carcinogenic signal is powerfully induced by double strand breakage. Thus, there seems to be a low possibility that SC1 and EC1-2 forcibly inhibit the cellular senescence mechanism as a cancer suppression process.

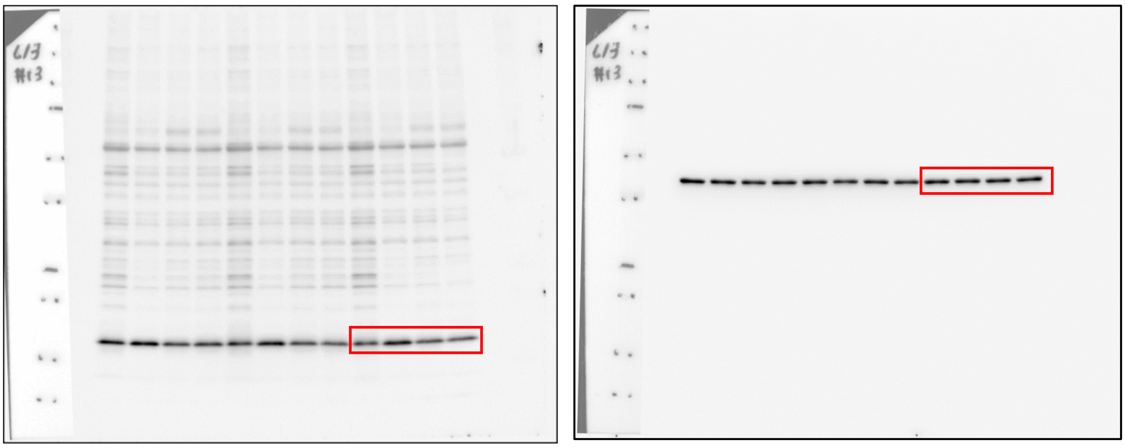

(a) (b)

Figure S2. Original gel images of western blot in Figure 3b. The gels images of p16 (a) and β-actin (b) are shown. Sample lane order from left is 1: Early passage, 2: Late passage, 3: SC1, 4: EC1-2, 5: Early passage, 6: Late passage, 7: SC1, 8: EC1-2, 9: Early passage, 10: Late passage, 11: SC1, 12: EC1-2 (include three independent experiments).
